# Design of an adaptable intrafascicular electrode (AIR) for selective nerve stimulation by model-based optimization

**DOI:** 10.1371/journal.pcbi.1011184

**Published:** 2023-05-25

**Authors:** Federico Ciotti, Andrea Cimolato, Giacomo Valle, Stanisa Raspopovic

**Affiliations:** Department of Health Sciences and Technology, Institute of Robotics and Intelligent Systems, ETH Zürich, Zürich, Switzerland; University of California Riverside, UNITED STATES

## Abstract

Peripheral nerve stimulation is being investigated as a therapeutic tool in several clinical scenarios. However, the adopted devices have restricted ability to obtain desired outcomes with tolerable off-target effects. Recent promising solutions are not yet employed in clinical practice due to complex required surgeries, lack of long-term stability, and implant invasiveness. Here, we aimed to design a neural interface to address these issues, specifically dimensioned for pudendal and sacral nerves to potentially target sexual, bladder, or bowel dysfunctions. We designed the adaptable intrafascicular radial electrode (AIR) through realistic computational models. They account for detailed human anatomy, inhomogeneous anisotropic conductance, following the trajectories of axons along curving and branching fascicles, and detailed biophysics of axons. The model was validated against available experimental data. Thanks to computationally efficient geometry-based selectivity estimations we informed the electrode design, optimizing its dimensions to obtain the highest selectivity while maintaining low invasiveness. We then compared the AIR with state-of-the-art electrodes, namely InterStim leads, multipolar cuffs and transversal intrafascicular multichannel electrodes (TIME). AIR, comprising a flexible substrate, surface active sites, and radially inserted intrafascicular needles, is designed to be implanted in a few standard steps, potentially enabling fast implants. It holds potential for repeatable stimulation outcomes thanks to its radial structural symmetry. When compared in-silico, AIR consistently outperformed cuff electrodes and InterStim leads in terms of recruitment threshold and stimulation selectivity. AIR performed similarly or better than a TIME, with quantified less invasiveness. Finally, we showed how AIR can adapt to different nerve sizes and varying shapes while maintaining high selectivity. The AIR electrode shows the potential to fill a clinical need for an effective peripheral nerve interface. Its high predicted performance in all the identified requirements was enabled by a model-based approach, readily applicable for the optimization of electrode parameters in any peripheral nerve stimulation scenario.

## Author summary

The potential of peripheral nerve stimulation in treating health conditions is limited by current technology, most importantly by the selectivity of the electrodes employed, i.e., their ability to obtain the desired clinical outcomes with limited collateral effects. Using highly realistic tridimensional models of the nerves and of the electrodes there implanted, we are able to estimate the outcome of stimulating with a specified electrode and protocol by simulating the behavior of each axon of the nerve. We used this computational framework to optimize key geometric parameters of a novel electrode design, specifically dimensioned for pudendal and sacral nerve stimulation for the potential treatment of bladder, bowel, and sexual dysfunctions. Within this in-silico framework, our electrode has shown promising results against state-of-the-art electrodes in all identified benchmarks, showing potential to meet the clinical need for a selective, quickly implantable electrode. The electrode optimization method we proposed can be applied in many other peripheral nerve stimulation scenarios for testing and optimizing electrode design parameters, aiding in the development of next-generation devices.

## Introduction

Peripheral nerve stimulation has been employed in clinics for decades for the treatment of several conditions such as severe depression and epilepsy by vagus nerve stimulation [1], and for the treatment of bladder dysfunctions by sacral neuromodulation [2]. However, the range of potential applications is much larger, since it extends to all functions which are regulated by the peripheral nervous system. Peripheral neurostimulation has shown positive outcomes in clinical trials in several applications such as sensory feedback restoration for amputees [3–5]. However, the efficacy and safety of neurostimulation are bounded by the current electrode technology. Devices used in clinical practice have a restricted ability to selectively obtain desired therapeutic effects with tolerable off-target effects, strongly limiting viable applications [6]. On the other hand, more invasive electrodes such as the transversal intrafascicular multichannel electrode (TIME) [7], have shown high performance in terms of selectivity in somatic nerves [8], but suffer from complex and long implant procedures [9]. With this work, we aimed to develop a neural interface with comparable or superior performance to current designs while limiting implant invasiveness and complexity.

**Fig 1:**
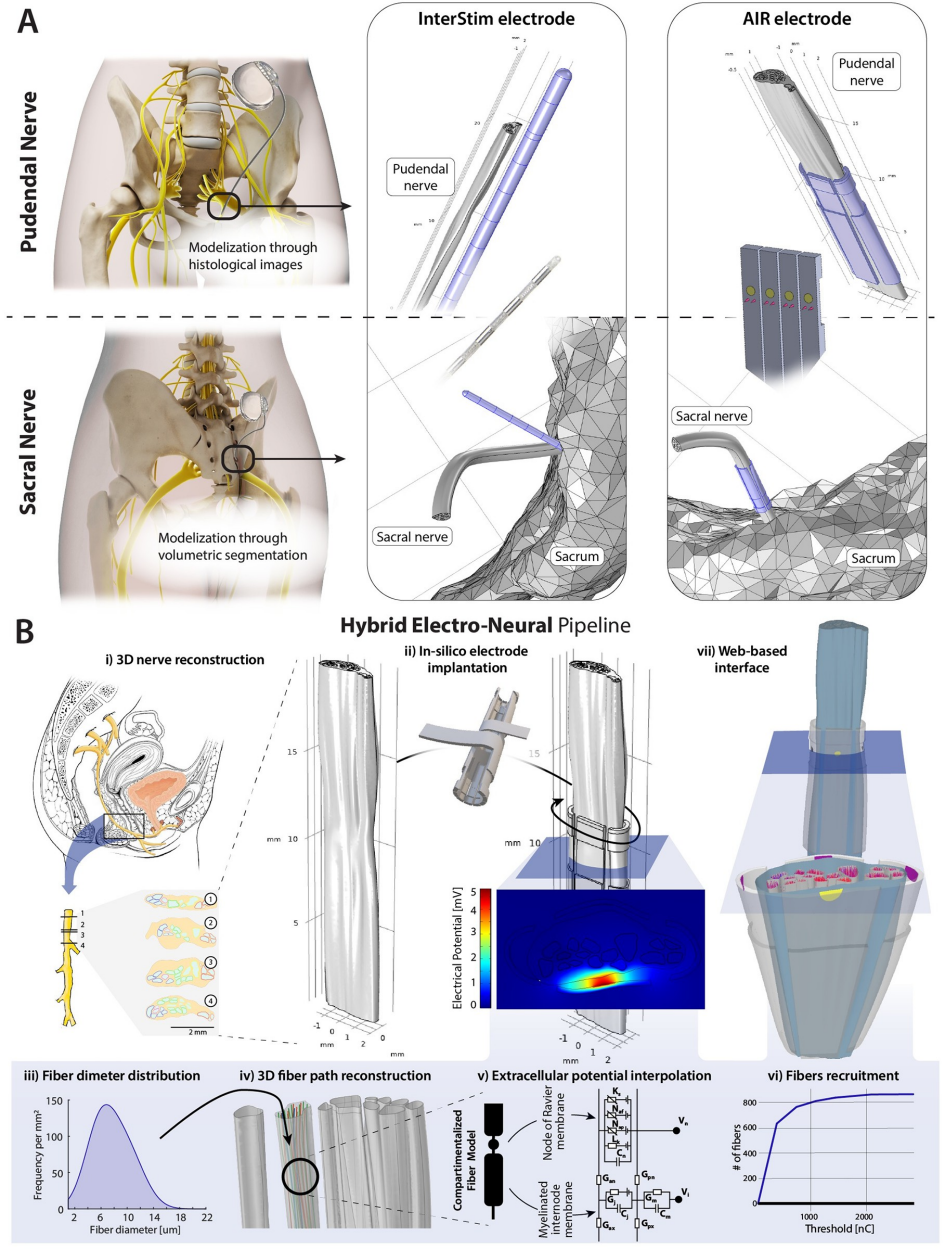
Proposed electrode placements and description of the hybrid modeling pipeline. **A**. The proposed placements of the electrodes are the pudendal nerve and the third sacral nerve, portrayed respectively on the top and bottom rows of the panel. On the left, a representation of the lead and implantable pulse generator placement. On the right, one exemplary modeled placement of InterStim and AIR electrodes on the target nerves (electrodes represented in blue). For the sacral nerve, the sacrum was also modeled for proper placement of the InterStim lead with respect to the sacral foramen. **B**. The hybrid modeling pipeline comprises: i) 3D nerve reconstructions made from histological information, modeled in Solidworks; ii) in-silico electrode implantation, integrating nerve and electrode geometries into a COMSOL model; iii) population of the fascicles with nerve fibers with diameters sampled from a predefined probability distribution; iv) 3D fiber path reconstruction through the solution of the curvilinear coordinates problem; v) solution of the current conservation problem with current injection by each active site and extracellular potential interpolation at each axonal compartment performed in COMSOL Multiphysics; vi) solution of the neural dynamics with NEURON to obtain fiber recruitment thresholds, and computation of recruitment curves; vii) analysis and display of results via MATLAB and a web-based interface using *three*.*js*.

We decided in particular to investigate the application of a novel peripheral electrode interface for pudendal and third sacral nerves, which bear the main control role in sexual, bladder, and bowel functions (Fig 1A). Treatment with peripheral neurostimulation has already shown promising results for pelvic dysfunctions and represents a great interest for future technological development due to their high prevalence and related costs. Indeed, sexual dysfunctions affect a very important share of the world population of female and male adults, a large portion of them related to genital arousal [10,11], which is known to be controllable by electrical nerve stimulation [12–17]. Oral drugs for erectile dysfunction have a limited efficacy [18], and for female arousal dysfunctions treatment options are very limited [19–21], showing the interest for a new therapeutic option. Stimulation of the pudendal nerve has been shown to trigger several effects such as motor responses of the external anal sphincter, external urethral sphincter, and intracavernous muscle; bladder contractions [22–24]; and bladder inhibition, exploited in clinical applications for the treatment of overactive bladder syndrome [25]. The mechanism of bladder inhibition is considered to be triggered by a spinal reflex [26], arguably mediated by larger myelinated afferents which are recruited at low stimulation amplitudes. The stimulation outcome is dependent on the electrode placement [22], therefore, the employment of selective electrodes may extend the clinical applications to other conditions such as sexual dysfunctions and on-demand control of bladder and bowel function. Since there exist somatotopic organization at the proposed implant level (e.g., clustering of fascicles in the pudendal nerve that will branch to inferior rectal nerve and dorsal genital nerve [27]), fascicular selectivity is necessary to modulate the function of target organs independently (e.g., control of bowel versus bladder function). Therefore, we chose it as a primary benchmark measure. However, because of the coexistence of different fiber types, mainly distinguishable by their diameter and carrying different functions, we also investigated how the selectivity by fiber diameter changes based on the electrode employed.

The space of parameters in the design of an electrode is extremely high-dimensional, therefore an iterative optimization through animal or human experimentation is unfeasible. For this reason, we decided to use hybrid computational models (Fig 1B) as a platform to identify an electrode design optimized in all its dimensions [28–30], an approach that is advocated for by several research groups [31–34]. Due to the high computational and human effort required to build highly detailed tridimensional models, we developed an optimization framework comprising a two-step approach. In the first step, a simplified geometric selectivity estimation allows to optimize each design parameter with minimal computational cost. The complete highly detailed computational model can be subsequently used to characterize the response to electrical stimulation down to single-axon resolution. The computational framework we developed can be applied in the future for the optimization of many peripheral nerve electrode designs.

Using this framework, we designed a novel neural interface, called adaptable intrafascicular radial electrode (AIR). We defined a mechanical design aiming to reduce invasiveness and implant complexity with respect to state-of-the-art intrafascicular electrodes. We then optimized its specific dimensions and configuration of active sites through an iterative process to maximize its selectivity. We chose to compare the AIR electrode with three other electrodes associated with different invasiveness levels: i) a quadripolar InterStim lead, as it is the clinical standard for sacral neuromodulation [2,35], and has also been proposed for pudendal neuromodulation [25,36]; ii) a multipolar cuff, to represent an extraneural approach typical of clinically used devices [6,30,37,38]; and iii) a TIME [7], an intrafascicular implant used in human clinical trials, which is highly selective on somatic nerves [8], and highly invasive [9]. Finally, we validated the model-predicted thresholds against experimental data available in literature for the InterStim implant on the sacral nerve.

## Results

### Framework validation

**Fig 2:**
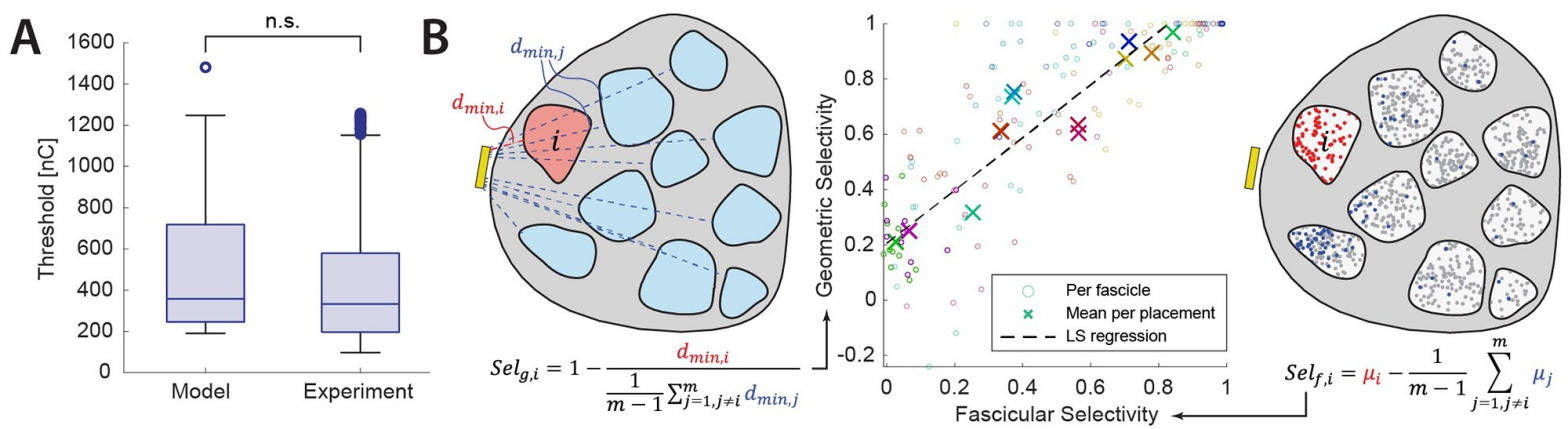
Validation of detailed and simplified computational models. **A**. Validation of the detailed computational model with literature available experimental data. The distribution of thresholds estimated by the model does not significantly differ from experimentally measured thresholds for the stimulation of the sacral nerve with an InterStim electrode [39] (Kolmogorov-Smirnov test, p > .05). **B**. Validation of the *geometric selectivity* as predictor of the mean *fascicular selectivity*. On the left, a qualitative representation of the *geometric selectivity*, where ***d***_***min*,*i***_ is the minimum distance (dashed line) from the active site (in yellow) to the target fascicle ***i*** (in red), and ***d***_***min*,*j***_ are the distances to each other fascicle (in blue). On the right, a qualitative representation of the *fascicular selectivity*, where ***μ***_***i***_ is the relative recruitment of the target fascicle ***i*** (in red), and ***μ***_***j***_ are the relative recruitments of each other fascicle (in blue). In the middle, the correlation plot between *fascicular selectivity*, estimated by the complete hybrid model, and *geometric selectivity*, calculated on geometric-only features, without performing any meshing or simulation. The least-square regression is drawn as dashed line. The *geometric selectivity* is a good predictor of the *fascicular selectivity* when averaged for all fascicles per electrode configuration (crosses) with an R^2^ = 0.92 (p < 0.0001), while it has lower predictive value for single fascicles (circles) with an R^2^ = 0.73 (p < 0.0001).

No significant differences were found between the distribution of thresholds predicted by the model for the InterStim implant on the sacral nerve with experimental thresholds (Kolmogorov-Smirnov test, p > .05) (Fig 2A). Comparable high-quality data for other nerve and electrode configurations were not available in literature. There exist a larger literature body regarding animal experimentation, but a comparison in absolute values is less meaningful because of large inter-species differences in neuroanatomy. For example, for the stimulation of feline pudendal nerve with an extraneural needle electrode are reported thresholds of 31 ± 19 nC [22], much lower than those reported in humans in a similar setup (130 ± 40 nC) [23].

### Electrode design optimization

**Fig 3:**
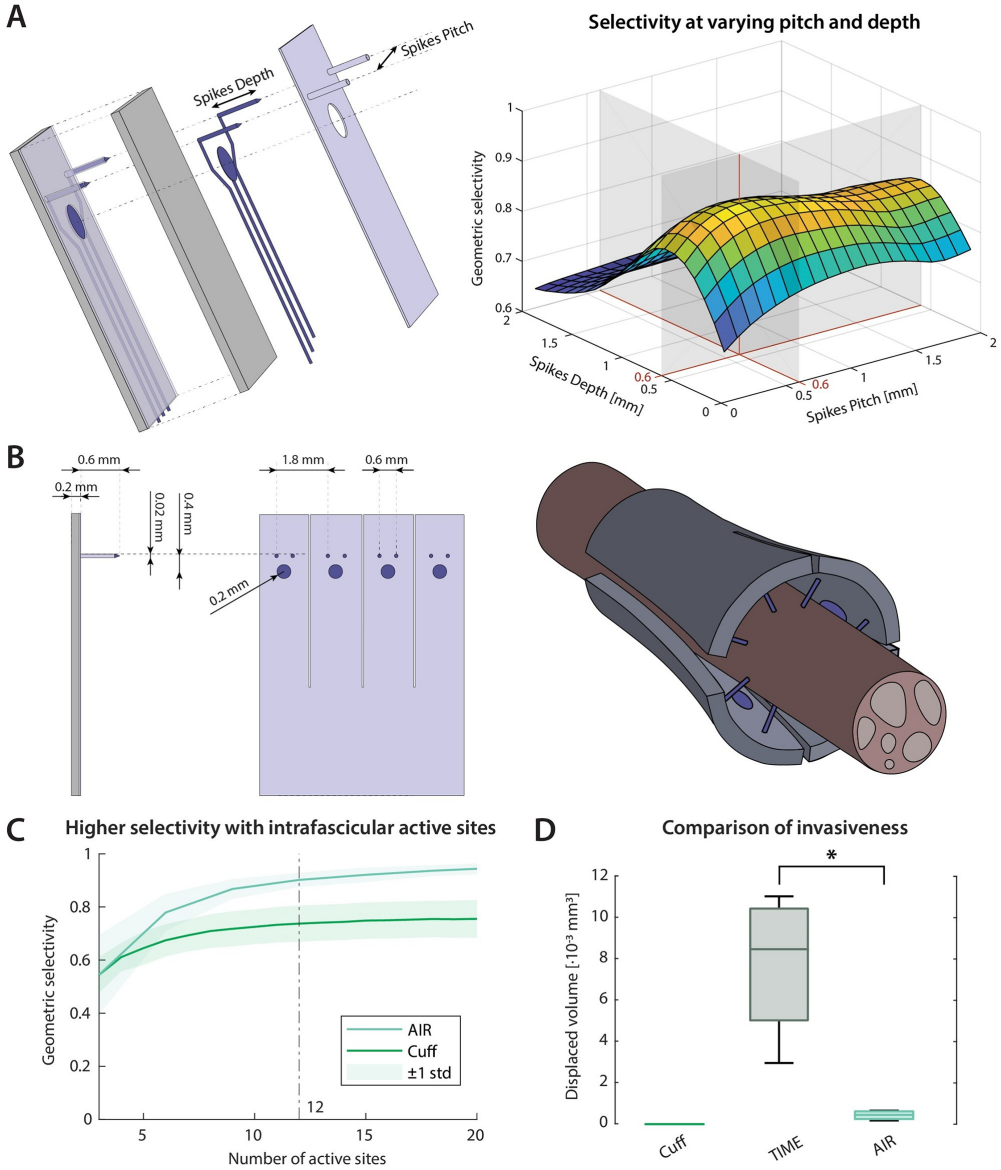
Optimization of the AIR electrode and estimation of its invasiveness. **A**. The optimization of the AIR electrode dimensions made by finding the absolute maximum of the geometric selectivity at varying pitch and depth of the spikes. **B**. The final design is shown with its dimensions and a representation of the electrode wrapped around a nerve. **C**. Selectivity at varying number of active sites, showing that cuff electrodes reach a plateau due to physical constraints while the AIR electrode can reach higher values of selectivity. For the AIR, the number of active sites was varied maintaining the ratio of 2 spikes/surface active site. The number of active sites chosen for the final design, 12, is highlighted. **D**. The invasiveness of the three implants is evaluated as the volume of endoneurium which is displaced during the electrode implant, showing that the AIR electrode causes lower fascicular damage than the other intrafascicular electrode.

The chosen electrode design consists of a cuff-like sleeve with a variable number of protrusions in the longitudinal direction of the nerve (referred to as *electrode heads*, Fig 3A). Each electrode head holds one surface active site and two sharp pillars (referred to as *spikes*), which are deinsulated at the tip, yielding two intrafascicular active sites. This structure was designed to allow a radial insertion of intrafascicular active sites, and to be able to adapt to varying nerve shapes and sizes (Fig 3B). Accordingly, we named it *adaptable intrafascicular radial (AIR) electrode*. The optimization of the AIR electrode dimensions was obtained by maximization of geometric selectivity which resulted in a spike pitch and a spike length of 0.6 mm (Fig 3A). The use of the geometric selectivity was justified by its high correlation with the mean fascicular selectivity computed by the complete hybrid model (R^2^ = 0.92, p < 0.001), and its negligible computational cost, requiring only the measurements of distances between fascicles and active sites (Fig 2B). We set the number of active sites to 12 (4 electrode heads), value at which the increment in selectivity per added active site was lower than 1% (Fig 3C). The choice of 2 spikes per electrode head was made by a similar compromise between selectivity and invasivity: increasing the from 1 to 2 spikes per head increased the geometric selectivity by 3% per added spike, while adding a third spike per head increase the selectivity by less than 1% per spike. Moreover, having two spikes per electrode head ensures its alignment to the nerve during the implant, aiding the radial insertion (see Fig 3B). We found that the optimized design had a higher selectivity asymptote than cuff electrodes (Fig 3C), arguably due to the more uniform distribution of active sites across the nerve, possibly justifying the higher invasiveness, which remains still lower than the one of TIMEs (Fig 3D).

### Recruitment thresholds and selectivities

**Fig 4:**
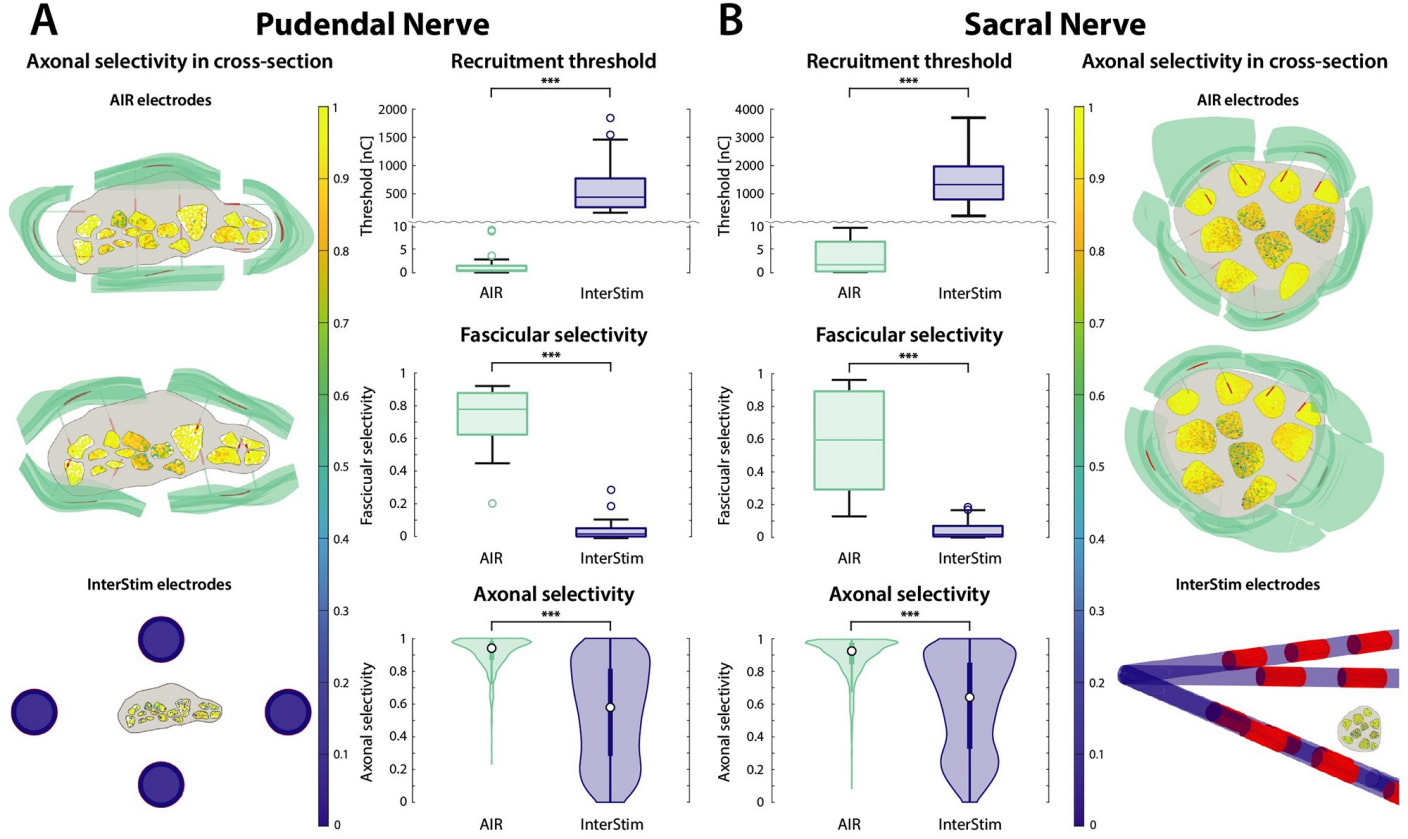
Recruitment thresholds, fascicular selectivity, and axonal selectivity of AIR and InterStim electrodes. Reported for **A**. Pudendal nerve, and **B**. Sacral nerve. For each electrode placement (2 for the AIR electrode, 4 for the InterStim), the fascicular threshold and selectivity were computed for 11 and 10 fascicles, and the axonal selectivity for 890 ± 16 and 5664 ± 30 axons (respectively for pudendal and sacral nerves). In the plots, for each electrode type, placements are grouped together. Results per single placement are reported in S2 Fig. The recruitment threshold plots show how the InterStim requires much higher injected charge and therefore energy to obtain the same recruitment. The selectivity plots show, for the InterStim, a median fascicular selectivity lower than 0.1 and axonal selectivity lower than 0.7, with the AIR electrode scoring statistically higher values. On the left of panel A, and on the right of panel B are drawn cross-sections of the nerve with axons color-coded by axonal selectivity, for the two modeled AIR placements separately of and for the four InterStim placements together. 3D models of the electrodes are overlaid on the cross-sections. The insulative substrates of the electrodes are represented in turquoise for the AIR and in blue for the InterStim, as for bar and violin plots. The active sites are represented in red.

The AIR electrode significantly outperformed the InterStim lead used in clinical practice in terms of recruitment thresholds, which are three orders of magnitude lower; fascicular selectivity; and axonal selectivity (p < .001) (Fig 4).

**Fig 5:**
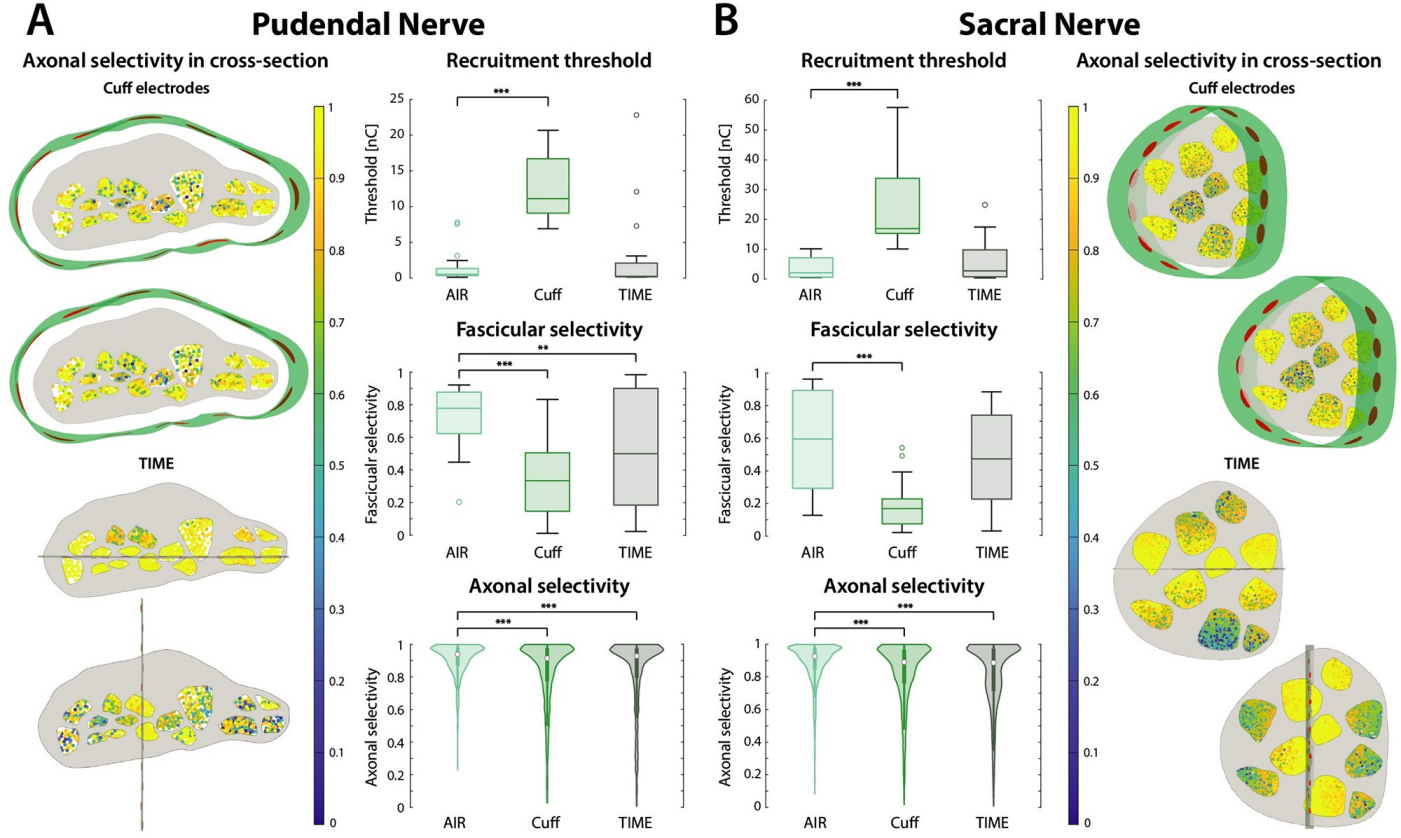
Recruitment thresholds, fascicular selectivity, and axonal selectivity of AIR electrode, cuff electrode, and TIME. Reported for **A**. Pudendal nerve, and **B**. Sacral nerve. For each electrode placement (2 for the AIR electrode, 2 for the cuff, and 2 for the TIME), the recruitment threshold and selectivity were computed for 11 and 10 fascicles, and the axonal selectivity for 890 ± 16 and 5664 ± 30 axons (respectively for pudendal and sacral nerves). In the plots, for each electrode type, placements are grouped together. Results per single placement are reported in S2 Fig. The AIR electrode shows significantly lower thresholds, higher fascicular selectivity, and higher axonal selectivity than the cuff electrode on both nerves. It shows significantly higher fascicular and axonal selectivity than the TIME on the pudendal nerve, and significantly higher axonal selectivity on the sacral nerve. The difference in thresholds between AIR and TIME was not significant on both nerves. Significant differences between TIME and cuff electrodes are not shown. On the left of panel A, and on the right of panel B are drawn cross-sections of the nerve with axons color-coded by axonal selectivity, for the two modeled cuff placements and for the two TIME placements. 3D models of the electrodes are overlaid on the cross-sections. The insulative substrates of the electrodes are represented in green for the cuff and in gray for the TIME, as for bar and violin plots. The active sites are represented in red.

We found that the AIR electrode performs significantly better than the cuff in terms of recruitment threshold, fascicular selectivity, and axonal selectivity, on both pudendal and sacral nerves (p < .001) (Fig 5). On the pudendal nerve, the AIR electrode shows significantly higher fascicular and axonal selectivity than the TIME (respectively p < .01 and p < .001), while the difference in thresholds is not significant (Fig 5A). On the sacral nerve, the AIR electrode outperforms the TIME electrode by all metrics, albeit significantly only in axonal selectivity (p < .001) (Fig 5B). In general, we observed a different behavior between pudendal and sacral nerve due to their different shape. We found that the flattened shape of the pudendal nerve allowed for better performance of the cuff electrode compared to implants on the rounder sacral nerve, due to a higher number of superficial fascicles. On the other side, the performance of TIMEs on pudendal nerves was highly dependent on proper placement, i.e., the outcome is less repeatable than with AIR and cuff electrodes, while on the sacral nerve its performance is less sensitive to its placement.

### Repeatability of stimulation outcome

The performance of the AIR and of the multipolar cuff in all three benchmark metrics was not significantly dependent (p > .05) on the modelled possible surgical placement (implemented in different positions with respect to the nerve), not on the pudendal nor on the sacral nerve. The axonal selectivity depended significantly on placement for both InterStim and TIME on pudendal nerve (resp. p < .01 and p < .001) and on sacral nerve (resp. p < .001 and p < .01). Recruitment thresholds depended significantly on placement for both InterStim and TIME on the pudendal nerve (resp. p < .001 and p < .05), but on the sacral nerve significantly only for the InterStim (p < .01). Fascicular selectivity depended significantly on surgical placement only for the TIME on the pudendal nerve (p < .001). Results for single placements with pairwise comparisons are reported in S2 Fig.

### Adaptability to variable nerve sizes

The adaptability study has shown that the AIR electrode is able to maintain a high selectivity level at increasing nerve size when the number of active sites is scaled accordingly. The TIME improves its selectivity at increasing nerve sizes when multiple electrodes are implanted in the same target nerve (panel B of S1 Fig). The AIR electrode outperforms other electrode designs for all nerve dimensions (p < .001), except for the larger tested nerve diameter (10.8 mm), for which the difference with 3 implanted TIMEs is not significant. Additionally, we found that the addition of surface active sites significantly improves the selectivity at all nerve dimensions (p < .001), with larger improvements (up to about 10%) for larger nerve sizes (panel A of S1 Fig).

### Selectivity by fiber diameter

**Fig 6:**
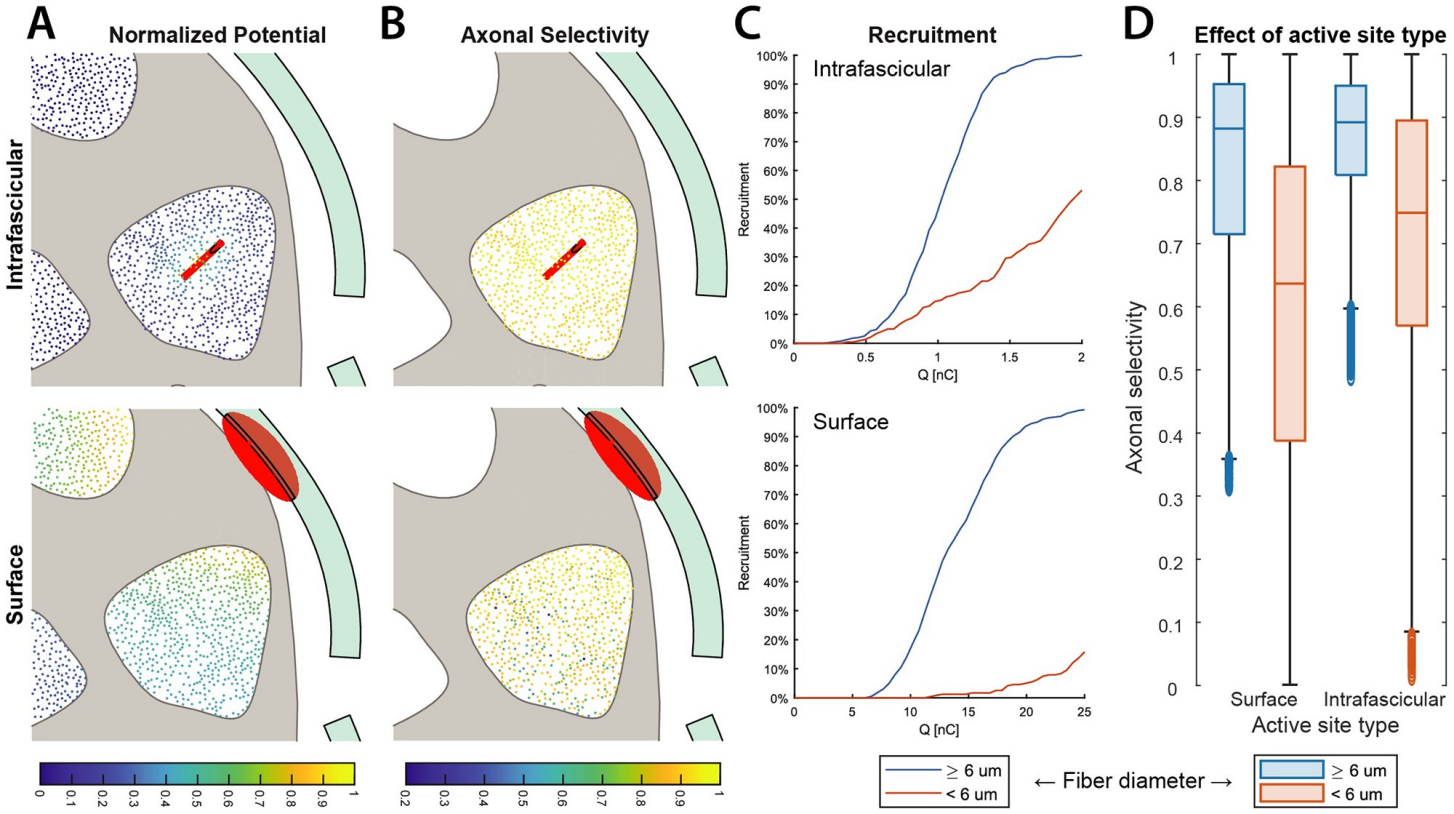
Effects of active site type on potential distribution, recruitment order, and selectivity. Difference in potential field (**A**) and therefore in fiber recruitment order (**C**) and in axonal selectivity (**B**) between axon diameter classes for an AIR intrafascicular versus extrafascicular active site. The active sites are represented in red 3D volumes. The black curve drawn within the active sites marks their intersection with the nerve cross-section plane. In (**D**) are reported the distributions of axonal selectivities for both placements of AIR on both nerves separately for extrafascicular and intrafascicular sites. Half intrafascicular active sites have been excluded from this analysis to avoid a bias due to different number of active sites in the two classes. The effects of active site type and fiber type on axonal selectivity have been analyzed by a two-way ANOVA with interaction terms and Tuckey’s test for multiple comparisons. Notably, the use of intrafascicular active sites increases the mean axonal selectivity for small axons by 0.11, p < .001, 95% CI [0.10, 0.12], while for large axons by only 0.04, p < .001, 95% CI [0.03, 0.05].

Moreover, the combination of intrafascicular and surface active sites of the AIR electrode may also enable differential strategies in recruiting axons of different diameter. Indeed, we observed how the order of fiber recruitment is fundamentally different between intrafascicular and extrafascicular stimulation (Fig 6C). For intrafascicular active sites, there is a strong dependance of recruitment threshold (and therefore axonal selectivity) on the distance to the active site, while for extrafascicular active sites the effect of axon diameter appears to be stronger (Fig 6A and 6B). This difference in recruitment order causes an increase of selectivity for smaller fiber diameters when using intrafascicular active sites (Fig 6C and 6D). A two-way ANOVA test with interaction terms highlighted a significantly higher increase of axonal selectivity for smaller diameters (0.11, p < .001, 95% CI [0.10, 0.12]) when using intrafascicular active sites, with respect to the increase of selectivity for larger axons (0.04, p < .001, 95% CI [0.03, 0.05], Fig 6D).

## Discussion

The high correlation between mean geometric and fascicular selectivity supports the choice of using the geometric selectivity as a computationally efficient target function when optimizing an electrode design for overall selectivity, and the use of the more realistic hybrid model when evaluating the detailed performance of an electrode at the fascicular or axonal level (Fig 2B). This allowed to optimize the AIR dimensions in a naïve grid-search with negligible computational costs. Performing the same procedure in the complete hybrid model would have required an impracticable computational and human effort. The evaluation of the final design in the complete model has shown promising results in terms of thresholds, selectivity, and invasiveness against other electrode designs.

The identified design, comprising multiple heads each holding one surface and two intrafascicular active sites, was meant to flexibly adapt to varying nerve shapes. Moreover, the split between heads and the inclusion of two spikes per electrode head guarantees the alignment of each head to the nerve surface during the implant (see Fig 3B), aiding a radial insertion of the spikes and reducing shear forces which may damage nerve and electrode. However, these observations need to be verified by future experiments. Intrafascicular electrodes typically require long and complex surgeries [9,40,41]. Ultimately, we foresee an implant procedure where the electrode substrate is wrapped around the nerve and fixed with suture, and then all electrode heads are tightened simultaneously with an external tightening strip, enabling simple and repeatable implants, but its feasibility is yet to be verified.

Lower recruitment thresholds indicate that the AIR electrode can be operated with lower power consumption and in a safer manner for the nervous tissue. This will allow for smaller batteries or longer time between re-implants or recharges via wireless power delivery systems, as well as more miniaturized electronics. Since there is no pre-existing knowledge regarding the amount of recruitment necessary to obtain clinically relevant effect, we decided to use the threshold to obtain 10% of fascicular recruitment as it is recurrent in literature [28,29,42–48]. However, because of the characteristic sigmoidal shape of recruitment curves, the recruitment increases very quickly around the 10% recruitment level. Indeed, when varying the target recruitment level between 5% and 15%, the thresholds predicted by our model remain quite stable (S4 Fig). Therefore, the presented results are mostly independent to the precise target recruitment level used for the determination of threshold.

The promising results in terms of both fascicular and axonal selectivity against neural interfaces currently used in research and clinical practice (Figs 4 and 5) suggest that the AIR design has higher therapeutic potential, since it indicates that the desired outcomes can be obtained thanks to reduced adverse effects [30].

Axonal selectivity, fascicular selectivity, and recruitment thresholds did not significantly depend on the modelled surgical placement for AIR and cuff electrodes, while for InterStim and TIME at least one of these metrics varied significantly. This result suggests that AIR and cuff electrodes would give more repeatable surgical outcomes, arguably thanks to their radial symmetry.

The lower invasiveness of the AIR electrode, quantified computationally as displaced fascicular volume, when compared to the TIME suggests that potentially it may be more acceptable for the use in clinical practice. However, the volume of penetration in the nerve tissue does not account for all aspects of invasivity, such as compression trauma, microhemorrhages, and possible mechanical breakages of the electrode, which would need to be evaluated in-vivo or through biomechanical models.

The AIR electrode has shown, in the simplified model, stable geometric selectivity values at increasing nerve sizes when the number of active sites is increased proportionally with the number of fascicles (S1), which suggest that it may be well applicable in larger nerves than pudendal and sacral nerves, e.g., it may be investigated for sensory restoration in lower limb amputees by stimulation of the sciatic nerve, which has an average diameter of 9 mm [49]. The TIME shows an increase of geometric selectivity when targeting larger nerves, at the cost of increasing the number of implanted electrodes. This strategy has been employed in the past [9,50], but it requires long and complex surgeries and is unlikely to be fully repeatable due to the precision constraints of the highly manual implant procedure.

The employed modeling framework has been validated against experimental results in similar circumstances [39]. To verify the plausibility of the present implementation we compared the estimated thresholds with experimental results, finding reasonable agreement. The highest quality data are available for the InterStim implant on the sacral nerve since it is a widely used clinical procedure. For this case, the predicted thresholds were not significantly different from the distribution found in literature [39] (Fig 2A), denoting that the modeled implants on the sacral nerve are plausible. The simulations respected experimental conditions such as electrode dimensions and waveform. To account for the natural variability of surgical electrode placement, we compared the experimental population with 12 simulated randomly sampled electrode placements, respecting the surgical implant through the third sacral foramen. Possible differences between model and reality include an incomplete representation of the tissues surrounding the nerve, a non-unanimous definition of recruitment threshold, and the missing representation of the electrical double layer between electrode and tissues. However, we considered reasonable to draw conclusions about the relative performance of different electrode designs even if systematic biases exist between model and reality. Moreover, the employed computational modeling framework has been shown in the past to properly replicate experimental results [28,40,43]. Nonetheless, the model predictions regarding the higher performance of the newly designed electrode need to be verified in a subsequent experimental study, which should include more extensive observations such as strength-duration curves for different fiber types. The use of flexible materials such as polyimide [7,51] for the manufacture of the AIR should allow it to conform to the nerve, but the experimental performance of the AIR may be affected by how electrode and nerve deform during the implant procedure. However, we accounted for possible variations by including a spacing of 189 ± 87 μm between surface active sites and nerve, which is likely larger than in real implants (Fig 4). Therefore, we expect lower thresholds and higher selectivities than estimated by the present models.

The differential behavior in recruitment order between intrafascicular and surface active sites can be explained by the different potential field generated, especially due to the presence the highly resistive perineurium, which causes more uniform distributions of potentials within the fascicles [52], reducing the effect of the distance on the recruitment threshold, emphasizing the effect of the axon diameter. Specifically, intrafascicular sites are able to obtain higher selectivity for smaller myelinated axons than extraneural sites. This behavior can be exploited, for example, when a different balance of large versus small diameter axon recruited is desired (Fig 6).

We recognize further limitations of this study, which include the lack of detailed histological information regarding sacral nerves, the use of fiber distributions from the sciatic nerve, and not modeling unmyelinated fibers. However, we believe that the amount of detail obtained from anatomical data and previous literature was sufficient to draw meaningful conclusions regarding the comparison of different electrode designs. The use of an arbitrary distribution of fiber diameters was also not expected to induce large differences in results regarding the comparison of electrodes. Avoiding modeling unmyelinated fibers seemed considerate since they typically show much higher recruitment thresholds at our chosen low frequency stimulation policy [53] and are not our stimulation target, which instead are mainly myelinated somatic axons in the pudendal nerve and preganglionic parasympathetic axons in the sacral nerve [54–56]. Finally, we did not account for the effect of fibrotic encapsulation with chronic use, which may be interesting to study by further modeling efforts [40].

We proposed a novel peripheral nerve interface which, on complex computational models, has shown potential improvements in terms of stimulation selectivity, implant repeatability, adaptability, and invasiveness, compared to devices currently used in research and clinical settings, and that the proposed AIR electrode design may prove an improvement over existing intrafascicular designs such as the TIME, as predicted by our modeling results.

The electrode design was optimized and tested on in-silico pudendal and sacral nerves with the aim of developing an effective neurostimulation device to treat sexual, bladder, and bowel dysfunctions. Nonetheless, the positive results in adaptability to varying nerve sizes makes it a candidate to target a wide range of nerves, from small autonomic nerves such as the vagus nerve, up to large somatic nerves as the sciatic nerve for sensory restoration.

The method to optimize electrode parameters we hereby proposed, consisting of a first grid search optimization on a simplified geometric selectivity index and test on a complete hybrid computational model, publicly available [57], can be moreover adopted for the identification and optimization of other peripheral electroneutral interfaces. Thus, our framework represents a novel cost-effective tool to design and optimize neural electrodes, avoiding long and costly iterative processes involving animal experimentation. Notably, the predictions regarding the performance need to be followed by experimental validation. Future steps will regard the evaluation of electrical and mechanical stability, safety and biocompatibility, and efficacy in obtaining desired physiological outcomes.

## Methods

### Novel design

The development of the design was guided by four main aims: *stimulation* s*electivity*, a requisite to obtain desired clinical outcomes effectively and safely, i.e., with minimal undesired effects; *implant* a*daptability*, the capability of the design to adapt to different nerve shapes and sizes; *low implant invasiveness*; and *implant repeatability*, the independence of the implant performance from specific surgical placement, e.g. rotations of the electrode around the nerve. The mechanical structure of the newly developed electrode entails a main flexible cuff-like substrate with several longitudinal extensions, each holding one surface active sites and two intraneural active sites on rigid parallel needles. This circumferential distribution of paired intrafascicular active sites was designed so that all needles can radially pierce the nerve simultaneously when the electrode is tightened around the nerve (Fig 3B). Its dimensions were optimized, by grid search, by maximizing an approximate geometrical estimation of fascicular selectivity with low computational cost (see *Metrics*) (Fig 3A). The design was optimized and compared to existing neural interfaces exploiting an in-silico platform already validated in previous works [28,29,43–47], enriched by a novel method to propagate the axons along the longitudinally developing fascicles and to parallelly apply the related inhomogeneous anisotropy (Fig 1).

### Hybrid computational model

#### Nerve model

The model of pudendal nerve was generated from four histological cross-sections along a 2 cm long ex-vivo human nerve sample [27] by assisted segmentation in MATLAB and 3D reconstruction in Solidworks by *Loft* features, obtaining a natural representation of branching and merging of fascicles, as previously described [44,46]. Given that for the human sacral nerve only analytical information (i.e. total area, area occupied by the endoneurium, and number of fascicles) was available [58], a synthetic nerve cross-section was generated with epineurium and fascicles made of random shapes obtained as convex hulls of a 2D Gaussian distribution of points, smoothed by periodic cubic spline interpolation. Since at the level of interest, within and leaving the sacral foramen, the sacral nerve has strong curvatures which are relevant for electrical modeling, the sacral nerve model was generated by sweeping the faux segmentation along a realistic trajectory extracted from the whole-body segmentation model *Jeduk* [59]. The sacrum was also extracted to later consider the presence of bone, which has a much lower conductance than soft tissues, and to have a physical reference for proper transforaminal insertion of InterStim leads. In both models, the perineurium was modeled with a thickness of 3% the equivalent diameter of each fascicle [60].

#### Electrodes

The InterStim lead was modeled according to Medtronic Model 3389 specifications [61], a quadripolar lead with a diameter of 1.27 mm and 3 mm long active sites spaced by 3 mm. The cuff was modeled as a thin polyimide film conformal to the nerve surface with 12 radially distributed active sites of 400 μm diameter, equally spaced (Fig 5). The choice of 12 active sites is justified by the saturation of selectivity observed with extraneural electrodes (see Fig 3C). The TIME was modeled according to TIME-3 specifications (due to the small nerves dimensions) with 12 active sites of 80 μm diameter, 6 per side, with at a pitch of 450 μm and a shift of half a pitch between the sides, for a total span of about 2.5 mm [62]. These dimensions were chosen so that the active sites would be distributed uniformly through a whole diameter of the target nerves (Fig 5). We set the number of active sites for the cuff electrode and the TIME equal to the number of active sites of the AIR electrode to ensure more meaningful comparisons of selectivity. The choice of the AIR electrode design and dimensions are reported in Fig 3. The diameter of the surface active sites of AIR and cuff electrodes was chosen to assure sufficient charge injection capacity, given that thresholds are expected to be one order of magnitude higher than for intrafascicular electrodes (Fig 5). In particular, their surface area was set to be 25 times larger than TIME active sites. The needle tips of the AIR were exposed by 200 μm to obtain a sufficient surface area for charge injection (2.5 larger than the TIME). We assumed the same contact material for all active sites (e.g., iridium oxide).

#### Volume conduction

The injection of current from each electrode’s active site was simulated in COMSOL Multiphysics 5.6 (COMSOL AB) by solving the current conservation law in form (1) with Ohm’s law in vector form (2), and by applying proper conductivity values to endoneurium (longitudinally 0.571 S/m, radially 0.0826 S/m), epineurium (0.0826 S/m), perineurium (0.00088 S/m), surrounding medium (saline solution, 2 S/m), and electrode substrate (10^−14^ S/m); and boundary conditions as described in [46]. The stimulation is applied by a current source boundary condition applied at the active site surface.


$$\nabla \cdot \sigma \nabla {\text{V}}_{E}=0$$ (1)

J=σE (2)

The endoneurium is anisotropic due to the presence of the axons. A novelty which we introduced in the hereby presented models regards the way axons are placed and how inhomogeneous anisotropy is simultaneously defined. We assumed that the trajectory of the axons could be compared to the streamlines of a diffusion phenomenon, therefore we created a diffusion physics where the ends of the fascicles on one side of the nerve are set as flow inlets, the other ends as outlets, and the fascicles’ lateral boundaries as walls. Thanks to the *Curvilinear Coordinates* feature of COMSOL, we could use the normalized gradient of the solution of the diffusion study as point-wise coordinate system where the anisotropy was then defined, and extract its streamlines as axon trajectories. This method assures that the anisotropy is always aligned with the axons and that the axons smoothly follow curvatures, changes of shape, branching, and merging of fascicles. Previous studies reported the use of similar methods for the definition of inhomogeneous anisotropic fields in the field of spinal cord stimulation [63,64]. However, they relied on spline interpolation for the generation of axon trajectories, which in case of peripheral nerves may not be applicable due to the typical substantial evolution of the branching fascicles along the nerve [65], and in general does not assure the alignment of the axons to the anisotropy field. To the best of our knowledge, a method for the generation of fiber trajectories intrinsically aligned with the anisotropy field has not been reported before.

#### Neural model

The population of axons was generated based on data available for the human sciatic nerve [66], due to the lack of comparable information for the target nerves. While the two nerves strongly differ in size, we expect to have similar myelinated fiber density and diameter distribution. Moreover, we found reasonable to assume that possible discrepancies would not affect the general conclusions regarding the comparison of different electrode designs. Axons were randomly distributed in the central cross-section of the nerves with density of 2.33 · 10^3^ mm^-2^, at a factor 5 subsampling than the reference human sciatic nerve, and with diameter following a mixing of two normal distributions with means 3.1 μm and 9.2 μm fit on the available human data [66], similarly to what has been done in previous studies [28,46,47,67]. The axon trajectories were generated starting from the axon centers previously placed in the central cross-section, iteratively extending them along the diffusion field resulting from the *Curvilinear Coordinates* study (see previous section) in both directions, until the first and last cross-sections of the modeled fascicles were reached. The fibers were modeled according to the McIntyre, Richardson, and Grill mammalian axon model [68] in NEURON 7.7.2 (Yale) [69]. A longitudinal random shift was applied to each initial node to avoid their alignment at the initial cross-section. The chosen stimulation policy was a cathodic square pulse with 50 μs width. For each axon, the extracellular potentials at each compartment along its trajectory were interpolated from the volume conduction solution and scaled iteratively to obtain the recruitment threshold with a bisection method. Extracellular potential distributions across the nerve for different electrode designs are reported in S3 Fig.

#### Validation

We compared the recruitment thresholds estimated by the model for the InterStim implant on the sacral nerve, for which we modeled 8 additional placements (n = 12), with experimental thresholds reported in literature (n = 48 implanted subjects) [39]. The experimental thresholds were obtained from figure 2A of [39]. Originally reported in terms of current, we converted them to charge by multiplication with the reported average value of pulse width (210.6 ± 11.6 μs). For each modeled placement, we chose the lowest threshold corresponding to the best active site, to replicate the optimal parameters choice made by the practitioner. Finally, we compared the distributions by a Kolmogorov-Smirnov test.

### Metrics

#### Recruitment threshold

To compare the amount of charge required to obtain desired functional outcomes, we computed a fascicle-specific *recruitment threshold* defined as the amount of charge necessary to recruit 10% of axons in the selected fascicle.

#### Selectivity

We evaluated the selectivity of a certain electrode in terms of the commonly used *fascicular selectivity* [45,47], defined in Eq (3), where *μ*_*i*_ is the relative recruitment of fascicle *i*; and in terms of *axonal selectivity*, which we hereby propose, to overcome limitations of previously used selectivity metrics which are detailed in S1 Text.


$$Sel_{f,i}=\mu_{i}-\frac{1}{m-1}\overset{m}{\underset{j=1,\;j\neq i}{\sum }}\mu_{j}$$ (3)

The *axonal selectivity* is defined in Eq (4), where *n*_*coll*,*i*_ is the minimum number of axons that are recruited together with the target axon *i* (i.e., the collateral activation with the best available stimulation policy), and *N* is the total number of axons. It quantifies the percentage of the nerve which must be recruited before reaching the target axon. It ranges from 0, when the target axon is the last to be recruited, to 1, when there exist a set of stimulation parameters which recruits the target axon individually.


$$Sel_{a,i}=1-\frac{n_{coll,i}}{N}$$ (4)

In case of monopolar stimulation with fixed waveform, such as ours, the best available stimulation policy is a stimulation performed at the recruitment threshold of the target axon using the active site chosen so that it simultaneously recruits the least number of other axons (with lower recruitment threshold than the target axon). *n*_*coll*,*i*_ can therefore be computed as in Eq (5), where *t*_*i*,*s*_ is the threshold to recruit axon *i* with active site *s*.


$$n_{coll,i}=\underset{s\in S}{\text{min}}\overset{N}{\underset{j=1,\;j\neq i}{\sum }}\left[t_{j,s}\leq t_{i,s}\right]$$ (5)

This metric can be adapted to consider groups of axons (e.g., fascicles or functional groups) by simple averaging, or by excluding the activation of other axons within the target group from the collateral activation, as detailed in S1 Text.

To optimize the electrode dimensions, it was not efficient to iteratively compute complete hybrid models since a change in the electrode geometry requires complete re-meshing and solving, meaning hours of computation for each iteration. Instead, we defined a new metric of selectivity which can be computed at extremely low cost, which we called *geometric selectivity* (Fig 2B). It estimates the fascicular selectivity based only on 2D geometric measures (6), where *d*_*min*,*i*_ is the minimum distance from fascicle *i* to the active site (0 if the active site is intrafascicular).


$$Sel_{g,i}=1-\frac{d_{min,i}}{\frac{1}{m-1}\sum_{j=1,\;j\neq i}^{m}d_{min,j}}$$ (6)

This metric is able to estimate the mean fascicular selectivity of a certain electrode configuration with an R^2^ = 0.92, allowing to optimize the dimensions of the electrode naively by a fast grid search, while per-fascicle is only moderately correlated, with an R^2^ = 0.73, showing that a more complete model is necessary to properly evaluate the performance of an electrode (Fig 2B). For all selectivity metrics, it is always reported the value corresponding to the best available active site for each target. The effects of electrode type and placement on thresholds and selectivities were evaluated by a two-way ANOVA where the effect of placement was nested in the effect of electrode type, with Bonferroni correction for multiple comparisons.

#### Invasiveness

The invasiveness of the implant was measured as the fascicular volume displaced when the electrode is inserted into the nerve. The results for the TIME electrode are likely a conservative estimate since the surgical procedure requires piercing a through-hole with a 150 μm diameter surgical needle prior to the electrode insertion [7]. As argued in *Discussion*, this metric allows to quantify only part of contributions to implant invasiveness.

#### Adaptability

The adaptability was estimated by generating synthetic nerve cross-sections from the four available pudendal nerve segmentations. The cross-sectional area was increased up to 9 times the mean original area. A kernel distribution was fit to the areas of the segmented fascicles and used to generate populations of fascicles in the synthetic cross-section. The distribution of the fascicular areas, the number of fascicles, and the number of active sites were scaled proportionally with the equivalent diameter of the nerve. The choice of scaling with the diameter and not with the area of the nerve was made to maintain: i) the density of active sites constant; ii) a constant ratio between number of active sites and number of fascicles; and iii) to represent the fact that larger nerves typically hold larger fascicles. Indeed, we obtained a mean ratio of single fascicle area to nerve diameter of 0.024 μm for the pudendal nerve, which is comparable with values found in literature for larger nerves, e.g. 0.026 μm for the sciatic nerve, which has a cross-sectional area on average 10 times larger than our pudendal nerve samples [70]. The results were obtained in terms of geometric selectivity and analyzed by a three-way ANOVA (effects of nerve diameter, electrode type, and random nerve sample) with Tukey’s test for multiple comparisons.

#### Repeatability

The repeatability–or invariance to placement–, was tested for the TIME model by inserting the electrode in two perpendicular placements. The InterStim leads were placed in four corners around the nerves, parallel to the pudendal nerve to represent an implant along the nerve in Alcock’s canal as described in literature [25], and slanted to the sacral nerve to simulate different insertion trajectories through the third sacral foramen [35]. The 12-polar cuff was modeled in two positions with a relative rotation of 15° (since the electrode is 12-fold radially symmetric, i.e., it matches the starting configuration when rotated by 30°). The AIR electrode, which is radially symmetric by 90° rotations, was modeled in two placements 45° apart. Repeatability was verified by evaluating the effect of placement on recruitment threshold, fascicular selectivity, and axonal selectivity by one-way ANOVA tests. Bonferroni correction was applied for pairwise comparisons.

#### Selectivity by fiber diameter

To study the differential effect of active site type of the AIR on different fiber types, we divided the fibers in two classes: small (diameter < 6 μm), and large (≥ 6 μm). We then compared the recruitment order and axonal selectivity obtained by selecting either all 4 surface active sites of the AIR electrode, or half of the 8 intrafascicular active sites. We performed this analysis on both pudendal and sacral nerves for both electrode placements. Finally, we analyzed the effects of active site type and fiber type on axonal selectivity by a two-way ANOVA with interaction terms and Tuckey’s test for multiple comparisons.

## Supporting information

S1 Text Supplementary methods. (PDF)

S1 Fig Adaptability to variable nerve sizes. **A**. Geometric selectivity of AIR with surface active sites and of AIR without surface active sites and the relative increment brought by the addition of surface active sites. Each electrode head holds two intrafascicular active sites, and one or no surface active site (green and gray lines respectively). **B**. Adaptability of AIR electrode, cuff electrode, and TIME to varying nerve size. For every nerve size, in both A. and B., it is reported mean ± std of geometric selectivity for 20 random synthetic nerve cross-sections. For both panels, a three-way ANOVA (effects of nerve diameter, electrode type, and random nerve sample) with Tukey’s test for multiple comparisons was performed. The star-signs label significant differences between the AIR and other electrode types across nerve sizes.
(PNG)

S2 Fig Effect of placement for each electrode type. The star-signs label significant differences within electrode type among placements, computed by one-way ANOVA tests with Bonferroni correction for multiple comparisons.
(PNG)

S3 Fig Distribution of electric potential interpolated at the fiber centers across the middle cross-section of the pudendal nerve. Obtained at a reference injected current of 1 mA for a selection of different active sites of InterStim, TIME, and AIR electrodes.
(PNG)

S4 Fig Distribution of thresholds for different target recruitment values. All electrode placements and fascicles are grouped. It can be observed how the relative performance of the four electrode types does not appreciably depend on the target recruitment level.
(PNG)

## References

[1] Beekwilder JP, Beems T. Overview of the Clinical Applications of Vagus Nerve Stimulation. Journal of Clinical Neurophysiology. 2010;27: 130–138. doi: 10.1097/WNP.0b013e3181d64d8a 20505378

[2] van Kerrebroeck PEV, van Voskuilen AC, Heesakkers JPFA, Lycklama á Nijholt AAB, Siegel S, Jonas U, et al. Results of Sacral Neuromodulation Therapy for Urinary Voiding Dysfunction: Outcomes of a Prospective, Worldwide Clinical Study. Journal of Urology. 2007;178: 2029–2034. doi: 10.1016/j.juro.2007.07.032 17869298

[3] Raspopovic S. Advancing limb neural prostheses. Science. 2020;370: 290–291. doi: 10.1126/science.abb1073 33060348

[4] Preatoni G, Valle G, Petrini FM, Raspopovic S. Lightening the Perceived Prosthesis Weight with Neural Embodiment Promoted by Sensory Feedback. Current Biology. 2021;31: 1065–1071.e4. doi: 10.1016/j.cub.2020.11.069 33417885

[5] Valle G, Saliji A, Fogle E, Cimolato A, Petrini FM, Raspopovic S. Mechanisms of neuro-robotic prosthesis operation in leg amputees. Science Advances. 2021;7: eabd8354. doi: 10.1126/sciadv.abd8354 33883127 PMC8059925

[6] Shulgach JA, Beam DW, Nanivadekar AC, Miller DM, Fulton S, Sciullo M, et al. Selective stimulation of the ferret abdominal vagus nerve with multi-contact nerve cuff electrodes. Sci Rep. 2021;11: 12925. doi: 10.1038/s41598-021-91900-1 34155231 PMC8217223

[7] Boretius T, Badia J, Pascual-Font A, Schuettler M, Navarro X, Yoshida K, et al. A transverse intrafascicular multichannel electrode (TIME) to interface with the peripheral nerve. Biosensors and Bioelectronics. 2010;26: 62–69. doi: 10.1016/j.bios.2010.05.010 20627510

[8] Badia J, Boretius T, Andreu D, Azevedo-Coste C, Stieglitz T, Navarro X. Comparative analysis of transverse intrafascicular multichannel, longitudinal intrafascicular and multipolar cuff electrodes for the selective stimulation of nerve fascicles. J Neural Eng. 2011;8: 036023. doi: 10.1088/1741-2560/8/3/036023 21558601

[9] Petrini FM, Bumbasirevic M, Valle G, Ilic V, Mijović P, Čvančara P, et al. Sensory feedback restoration in leg amputees improves walking speed, metabolic cost and phantom pain. Nature Medicine. 2019;25: 1356–1363. doi: 10.1038/s41591-019-0567-3 31501600

[10] Lewis RW, Fugl-Meyer KS, Corona G, Hayes RD, Laumann EO, M ED Jr, et al. Definitions/Epidemiology/Risk Factors for Sexual Dysfunction. The Journal of Sexual Medicine. 2010;7: 1598–1607. doi: 10.1111/j.1743-6109.2010.01778.x 20388160

[11] Kubin M, Wagner G, Fugl-Meyer AR. Epidemiology of erectile dysfunction. Int J Impot Res. 2003;15: 63–71. doi: 10.1038/sj.ijir.3900949 12605242

[12] Eckhard C. Untersuchungen über die Erektion beim Hunde. Beiträge zur Anatomie und Physiologie. 1863;3: 123–166.

[13] Trigo-Rocha F, Aronson WJ, Hohenfellner M, Ignarro LJ, Rajfer J, Lue TF. Nitric oxide and cGMP: mediators of pelvic nerve-stimulated erection in dogs. American Journal of Physiology-Heart and Circulatory Physiology. 1993;264: H419–H422. doi: 10.1152/ajpheart.1993.264.2.H419 8383456

[14] Hox M, Mann-Gow T, Lund L, Zvara P. Cavernous Nerve Stimulation and Recording of Intracavernous Pressure in a Rat. JoVE (Journal of Visualized Experiments). 2018; e56807. doi: 10.3791/56807 29733311 PMC6100706

[15] Sturny M, Karakus S, Fraga-Silva R, Stergiopulos N, Burnett AL. Low-Intensity Electrostimulation Enhances Neuroregeneration and Improves Erectile Function in a Rat Model of Cavernous Nerve Injury. The Journal of Sexual Medicine. 2022;19: 686–696. doi: 10.1016/j.jsxm.2022.02.004 35288047

[16] Vachon P, Simmerman N, Zahran AR, Carrier S. Increases in clitoral and vaginal blood flow following clitoral and pelvic plexus nerve stimulations in the female rat. Int J Impot Res. 2000;12: 53–57. doi: 10.1038/sj.ijir.3900480 10982313

[17] Kim SW, Jeong S-J, Munarriz R, Kim NN, Goldstein I, Traish AM. Role of the nitric oxide-cyclic GMP pathway in regulation of vaginal blood flow. International Journal of Impotence Research. 2003;15: 355–361. doi: 10.1038/sj.ijir.3901038 14562137

[18] Shamloul R, Ghanem H. Erectile dysfunction. The Lancet. 2013;381: 153–165. doi: 10.1016/S0140-6736(12)60520-0 23040455

[19] Basson R, Wierman ME, Van Lankveld J, Brotto L. REPORTS: Summary of the Recommendations on Sexual Dysfunctions in Women. The Journal of Sexual Medicine. 2010;7: 314–326. doi: 10.1111/j.1743-6109.2009.01617.x 20092441

[20] Graham CA. The DSM Diagnostic Criteria for Female Sexual Arousal Disorder. Arch Sex Behav. 2010;39: 240–255. doi: 10.1007/s10508-009-9535-1 19777335

[21] McCabe MP, Sharlip ID, Atalla E, Balon R, Fisher AD, Laumann E, et al. Definitions of Sexual Dysfunctions in Women and Men: A Consensus Statement From the Fourth International Consultation on Sexual Medicine 2015. The Journal of Sexual Medicine. 2016;13: 135–143. doi: 10.1016/j.jsxm.2015.12.019 26953828

[22] Yoo PB, Grill WM. Minimally-invasive electrical stimulation of the pudendal nerve: A pre-clinical study for neural control of the lower urinary tract. Neurourology and Urodynamics. 2007;26: 562–569. doi: 10.1002/nau.20376 17262838

[23] Vodušek DB, Plevnik S, Vrtačnik P, Janež J. Detrusor inhibition on selective pudendal nerve stimulation in the perineum. Neurourology and Urodynamics. 1987;6: 389–393. doi: 10.1002/nau.1930060506

[24] Schmidt MH, Schmidt HS. The Ischiocavernosus and Bulbospongiosus Muscles in Mammalian Penile Rigidity. Sleep. 1993;16: 171–183. doi: 10.1093/sleep/16.2.171 8446838

[25] Spinelli M, Malaguti S, Giardiello G, Lazzeri M, Tarantola J, VD Hombergh U. A new minimally invasive procedure for pudendal nerve stimulation to treat neurogenic bladder: Description of the method and preliminary data. Neurourology and Urodynamics. 2005;24: 305–309. doi: 10.1002/nau.20118 15977260

[26] McGee MJ, Danziger ZC, Bamford JA, Grill WM. A spinal GABAergic mechanism is necessary for bladder inhibition by pudendal afferent stimulation. American Journal of Physiology-Renal Physiology. 2014;307: F921–F930. doi: 10.1152/ajprenal.00330.2014 25143456 PMC4200298

[27] Gustafson KJ, Zelkovic PF, Feng AH, Draper CE, Bodner DR, Grill WM. Fascicular anatomy and surgical access of the human pudendal nerve. World J Urol. 2005;23: 411–418. doi: 10.1007/s00345-005-0032-4 16333625

[28] Zelechowski M, Valle G, Raspopovic S. A computational model to design neural interfaces for lower-limb sensory neuroprostheses. J NeuroEngineering Rehabil. 2020;17: 24. doi: 10.1186/s12984-020-00657-7 32075654 PMC7029520

[29] Romeni S, Valle G, Mazzoni A, Micera S. Tutorial: a computational framework for the design and optimization of peripheral neural interfaces. Nat Protoc. 2020;15: 3129–3153. doi: 10.1038/s41596-020-0377-6 32989306

[30] Aristovich K, Donega M, Fjordbakk C, Tarotin I, Chapman CAR, Viscasillas J, et al. Model-based geometrical optimisation and in vivo validation of a spatially selective multielectrode cuff array for vagus nerve neuromodulation. Journal of Neuroscience Methods. 2021;352: 109079. doi: 10.1016/j.jneumeth.2021.109079 33516735

[31] Capogrosso M, Lempka SF. A computational outlook on neurostimulation. Bioelectronic Medicine. 2020;6: 10. doi: 10.1186/s42234-020-00047-3 32490037 PMC7247210

[32] Musselman ED, Cariello JE, Grill WM, Pelot NA. ASCENT (Automated Simulations to Characterize Electrical Nerve Thresholds): A pipeline for sample-specific computational modeling of electrical stimulation of peripheral nerves. PLOS Computational Biology. 2021;17: e1009285. doi: 10.1371/journal.pcbi.1009285 34492004 PMC8423288

[33] Raspopovic S. Neurorobotics for neurorehabilitation. Science. 2021;373: 634–635. doi: 10.1126/science.abj5259 34353946

[34] Grill WM. Model-based analysis and design of waveforms for efficient neural stimulation. In: Bestmann S, editor. Progress in Brain Research. Elsevier; 2015. pp. 147–162. 10.1016/bs.pbr.2015.07.031 PMC4772858 26541380

[35] Williams ER, Siegel SW. Procedural techniques in sacral nerve modulation. Int Urogynecol J. 2010;21: 453–460. doi: 10.1007/s00192-010-1280-4 20972542

[36] Peters KM, Feber KM, Bennett RC. Sacral versus pudendal nerve stimulation for voiding dysfunction: A prospective, single-blinded, randomized, crossover trial. Neurourology and Urodynamics. 2005;24: 643–647. doi: 10.1002/nau.20174 16178000

[37] Horn CC, Ardell JL, Fisher LE. Electroceutical Targeting of the Autonomic Nervous System. Physiology. 2019;34: 150–162. doi: 10.1152/physiol.00030.2018 30724129 PMC6586833

[38] Jayaprakash N, Toth V, Song W, Vardhan A, Levy T, Tomaio J, et al. Organ- and function-specific anatomical organization and bioelectronic modulation of the vagus nerve. bioRxiv; 2022. p. 2022.03.07.483266. doi: 10.1101/2022.03.07.483266

[39] Blok B, Van Kerrebroeck P, de Wachter S, Ruffion A, Van der Aa F, Jairam R, et al. Programming settings and recharge interval in a prospective study of a rechargeable sacral neuromodulation system for the treatment of overactive bladder. Neurourology and Urodynamics. 2018;37: S17–S22. doi: 10.1002/nau.23476 29336058

[40] Valle G, Aiello G, Ciotti F, Cvancara P, Martinovic T, Kravic T, et al. Multifaceted understanding of human nerve implants to design optimized electrodes for bioelectronics. Biomaterials. 2022; 121874. doi: 10.1016/j.biomaterials.2022.121874 36334353

[41] Strauss I, Niederhoffer T, Giannotti A, Panarese AM, Bernini F, Gabisonia K, et al. Q-PINE: A quick to implant peripheral intraneural electrode. J Neural Eng. 2020;17: 066008. doi: 10.1088/1741-2552/abc52a 33108764

[42] Badi M, Wurth S, Scarpato I, Roussinova E, Losanno E, Bogaard A, et al. Intrafascicular peripheral nerve stimulation produces fine functional hand movements in primates. Science Translational Medicine. 2021;13: eabg6463. doi: 10.1126/scitranslmed.abg6463 34705521

[43] Raspopovic S, Capogrosso M, Badia J, Navarro X, Micera S. Experimental Validation of a Hybrid Computational Model for Selective Stimulation Using Transverse Intrafascicular Multichannel Electrodes. IEEE Transactions on Neural Systems and Rehabilitation Engineering. 2012;20: 395–404. doi: 10.1109/TNSRE.2012.2189021 22481834

[44] Cimolato A, Ciotti F, Kljajić J, Valle G, Raspopovic S. Symbiotic electroneural and musculoskeletal framework to encode proprioception via neurostimulation: ProprioStim. iScience. 2023;26: 106248. doi: 10.1016/j.isci.2023.106248 36923003 PMC10009292

[45] Raspopovic S, Capogrosso M, Micera S. A Computational Model for the Stimulation of Rat Sciatic Nerve Using a Transverse Intrafascicular Multichannel Electrode. IEEE Transactions on Neural Systems and Rehabilitation Engineering. 2011;19: 333–344. doi: 10.1109/TNSRE.2011.2151878 21693427

[46] Ciotti F, Valle G, Pedrocchi A, Raspopovic S. A Computational Model of the Pudendal Nerve for the Bioelectronic Treatment of Sexual Dysfunctions. 2021 10th International IEEE/EMBS Conference on Neural Engineering (NER). 2021. pp. 267–270.

[47] Raspopovic S, Petrini FM, Zelechowski M, Valle G. Framework for the Development of Neuroprostheses: From Basic Understanding by Sciatic and Median Nerves Models to Bionic Legs and Hands. Proceedings of the IEEE. 2017. doi: 10.1109/JPROC.2016.2600560

[48] Polasek KH, Hoyen HA, Keith MW, Tyler DJ. Human Nerve Stimulation Thresholds and Selectivity Using a Multi-contact Nerve Cuff Electrode. IEEE Transactions on Neural Systems and Rehabilitation Engineering. 2007;15: 76–82. doi: 10.1109/TNSRE.2007.891383 17436879

[49] Gustafson KJ, Grinberg Y, Joseph S, Triolo RJ. Human distal sciatic nerve fascicular anatomy: Implications for ankle control using nerve-cuff electrodes. JRRD. 2012;49: 309. doi: 10.1682/jrrd.2010.10.0201 22773531

[50] Petrini FM, Valle G, Bumbasirevic M, Barberi F, Bortolotti D, Cvancara P, et al. Enhancing functional abilities and cognitive integration of the lower limb prosthesis. Science Translational Medicine. 2019;11. doi: 10.1126/scitranslmed.aav8939 31578244

[51] Steins H, Mierzejewski M, Brauns L, Stumpf A, Kohler A, Heusel G, et al. A flexible protruding microelectrode array for neural interfacing in bioelectronic medicine. Microsyst Nanoeng. 2022;8: 1–15. doi: 10.1038/s41378-022-00466-z 36568135 PMC9772315

[52] Pelot NA, Behrend CE, Grill WM. On the parameters used in finite element modeling of compound peripheral nerves. J Neural Eng. 2018;16: 016007. doi: 10.1088/1741-2552/aaeb0c 30507555 PMC7309635

[53] Chang Y-C, Cracchiolo M, Ahmed U, Mughrabi I, Gabalski A, Daytz A, et al. Quantitative estimation of nerve fiber engagement by vagus nerve stimulation using physiological markers. Brain Stimulation. 2020;13: 1617–1630. doi: 10.1016/j.brs.2020.09.002 32956868

[54] Steers WD. Neural pathways and central sites involved in penile erection: neuroanatomy and clinical implications. Neuroscience & Biobehavioral Reviews. 2000;24: 507–516. doi: 10.1016/s0149-7634(00)00019-1 10880817

[55] Giraldi A, Marson L, Nappi R, Pfaus J, Traish AM, Vardi Y, et al. Physiology of female sexual function: animal models. J Sex Med. 2004;1: 237–253. doi: 10.1111/j.1743-6109.04037.x 16422954

[56] Jang HS, Cho KH, Hieda K, Kim JH, Murakami G, Abe S, et al. Composite nerve fibers in the hypogastric and pelvic splanchnic nerves: an immunohistochemical study using elderly cadavers. Anat Cell Biol. 2015;48: 114. doi: 10.5115/acb.2015.48.2.114 26140222 PMC4488639

[57] Ciotti F, Cimolato A, Valle G, Raspopovic S. Hybrid Electro-Neural Simulation Platform—Pudendal and sacral nerve models. Zenodo; 2023. doi: 10.5281/zenodo.7805759

[58] Ebraheim NA, Lu J, Yang H, Huntoon M, Yeasting RA. Lumbosacral Plexus: A Histological Study. CTO. 1997;158: 274–278. doi: 10.1159/000147940 9416358

[59] IT’IS Foundation. Jeduk cV3.1. 2019 [cited 4 Sep 2020].

[60] Grinberg Y, Schiefer MA, Tyler DJ, Gustafson KJ. Fascicular Perineurium Thickness, Size, and Position Affect Model Predictions of Neural Excitation. IEEE Transactions on Neural Systems and Rehabilitation Engineering. 2008;16: 572–581. doi: 10.1109/TNSRE.2008.2010348 19144589 PMC2918421

[61] Medtronic. InterStim Product Catalogue. 2020. https://europe.medtronic.com/content/dam/medtronic-com/xd-en/hcp/documents/pelvic-health/interstim-product-catalogue.pdf.

[62] Čvančara P, Valle G, Müller M, Guiho T, Hiairrassary A, Petrini F, et al. On the Reliability of Chronically Implanted Thin-Film Electrodes in Human Arm Nerves for Neuroprosthetic Applications. bioRxiv; 2019. p. 653964. doi: 10.1101/653964

[63] Greiner N, Barra B, Schiavone G, Lorach H, James N, Conti S, et al. Recruitment of upper-limb motoneurons with epidural electrical stimulation of the cervical spinal cord. Nat Commun. 2021;12: 435. doi: 10.1038/s41467-020-20703-1 33469022 PMC7815834

[64] Rowald A, Komi S, Demesmaeker R, Baaklini E, Hernandez-Charpak SD, Paoles E, et al. Activity-dependent spinal cord neuromodulation rapidly restores trunk and leg motor functions after complete paralysis. Nat Med. 2022;28: 260–271. doi: 10.1038/s41591-021-01663-5 35132264

[65] Stewart JD. Peripheral nerve fascicles: Anatomy and clinical relevance. Muscle & Nerve. 2003;28: 525–541. doi: 10.1002/mus.10454 14571454

[66] Garven HSD, Gairns FW, Smith G. The Nerve Fibre Populations of the Nerves of the Leg in Chronic Occlusive Arterial Disease in Man. Scott Med J. 1962;7: 250–265. doi: 10.1177/003693306200700602 13897116

[67] Schiefer MA, Triolo RJ, Tyler DJ. A Model of Selective Activation of the Femoral Nerve With a Flat Interface Nerve Electrode for a Lower Extremity Neuroprosthesis. IEEE Trans Neural Syst Rehabil Eng. 2008;16: 195–204. doi: 10.1109/TNSRE.2008.918425 18403289 PMC2920206

[68] McIntyre CC, Richardson AG, Grill WM. Modeling the excitability of mammalian nerve fibers: influence of afterpotentials on the recovery cycle. J Neurophysiol. 2002;87: 995–1006. doi: 10.1152/jn.00353.2001 11826063

[69] Carnevale NT, Hines ML. The NEURON Book. Cambridge University Press; 2006.

[70] S Ugrenovic Z, I Jovanovic D, B Stefanović D. Microanatomical structure of the human sciatic nerve. Surg Radiol Anat. 2008;30: 619–626. doi: 10.1007/s00276-008-0386-6 18648720

