# Supplementary material for: Design of an adaptable intrafascicular electrode (AIR) for selective nerve stimulation by model-based optimization
Source: PLoS Comput Biol. 2023 May 25;19(5):e1011184. doi: 10.1371/journal.pcbi.1011184 (PMC10246853; doi:10.1371/journal.pcbi.1011184)
Supplement: S1 Text — (PDF) [file pcbi.1011184.s001.pdf]

S1 Text. Supplementary Methods

Traditionally, the stimulation selectivity of a certain electrode has been evaluated in terms of per-fascicle so-called spatial ($Sel_{S,i} = \frac{n_i}{N_i} - \frac{1}{m-1}\sum_{j=1,j\neq i}^{m}\frac{n_j}{N_j}$), or functional selectivity ($Sel_{F,i} = \frac{n_i}{\sum_{j=1}^{m} n_j}$) [1,2]. The main drawbacks of spatial and functional selectivity are that **1.** Their value depends on a specific set of stimulation parameters, e.g., are calculated at a specific injected charge level. For example, the value of the spatial selectivity will never exceed the recruitment level of the target fascicle, with the possibility of reaching 1 only with the unlikely intent of recruiting 100% of the target fascicle. **2.** The absolute value of functional selectivity is poorly interpretable: it is unstable around recruitment threshold, i.e. equals 1 if the very first recruited fibers belong to the target fascicle, 0 otherwise, and varying as soon as collateral recruitment appears quickly reaching a horizontal asymptote at increasing injected charge levels given by the ratio $\frac{N_i}{\sum_{j=1}^{m} N_j}$. **3.** The spatial selectivity heavily depends on the absolute number of fascicles, so it is not stable between different nerves or after natural merging and branching of fascicles. **4.** They only carry fascicle-level information, losing information regarding the variability within the fascicle due to spatial effects or distribution of axon diameters.

The formulation of *axonal selectivity* ($Sel_i = 1 - \frac{n_{coll,i}}{N}$) can be adapted to consider groups of axons (e.g., fascicles or functional groups) by simple averaging, or by modifying the definition to exclude activation within the target group from collateral activation:

$$Sel_G = \frac{1}{N_G}\sum_{i=1}^{N_G} 1 - \frac{n_{coll,G_i,\neg G}}{N}$$

where G_i is the i-th axon in group G of size N_G, and $n_{coll,G_i,\neg G}$ is the minimum number of axons *outside of* G that are recruited together with G_i. A value of 100% indicates that each axon in group G can be recruited without any activation outside of group G, while a value of 0 indicates that the axons in group G are always recruited last.

References

1. Raspopovic S, Petrini FM, Zelechowski M, Valle G. Framework for the Development of Neuroprostheses: From Basic Understanding by Sciatic and Median Nerves Models to Bionic Legs and Hands. Proceedings of the IEEE. 2017. doi:10.1109/JPROC.2016.2600560

2. Raspopovic S, Capogrosso M, Micera S. A Computational Model for the Stimulation of Rat Sciatic Nerve Using a Transverse Intrafascicular Multichannel Electrode. IEEE Transactions on Neural Systems and Rehabilitation Engineering. 2011;19: 333–344. doi:10.1109/TNSRE.2011.2151878
