# Supplementary figures and images for: Design of an adaptable intrafascicular electrode (AIR) for selective nerve stimulation by model-based optimization
Source: PLoS Comput Biol. 2023 May 25;19(5):e1011184. doi: 10.1371/journal.pcbi.1011184 (PMC10246853; doi:10.1371/journal.pcbi.1011184)

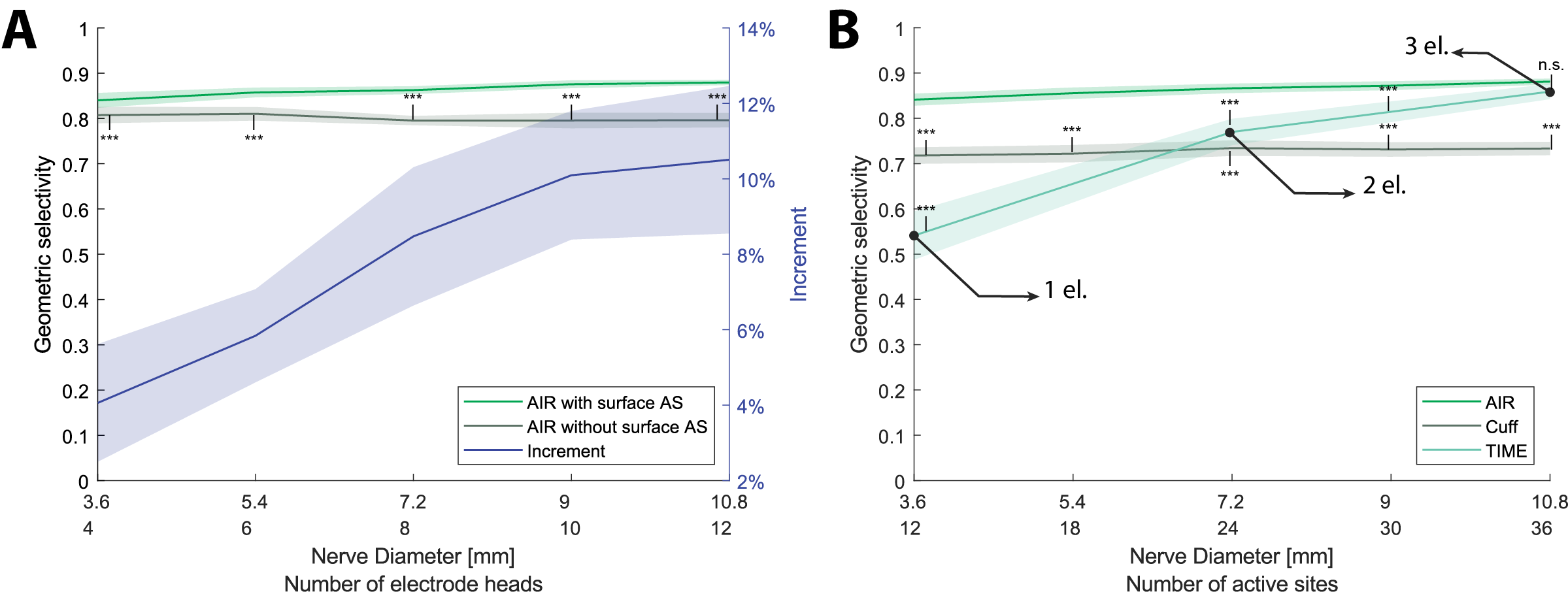

Supplement: S1 Fig — A. Geometric selectivity of AIR with surface active sites and of AIR without surface active sites and the relative increment brought by the addition of surface active sites. Each electrode head holds two intrafascicular active sites, and one or no surface active site (green and gray lines respectively). B. Adaptability of AIR electrode, cuff electrode, and TIME to varying nerve size. For every nerve size, in both A. and B., it is reported mean ± std of geometric selectivity for 20 random synthetic nerve cross-sections. For both panels, a three-way ANOVA (effects of nerve diameter, electrode type, and random nerve sample) with Tukey’s test for multiple comparisons was performed. The star-signs label significant differences between the AIR and other electrode types across nerve sizes. (PNG) [file pcbi.1011184.s002.png]

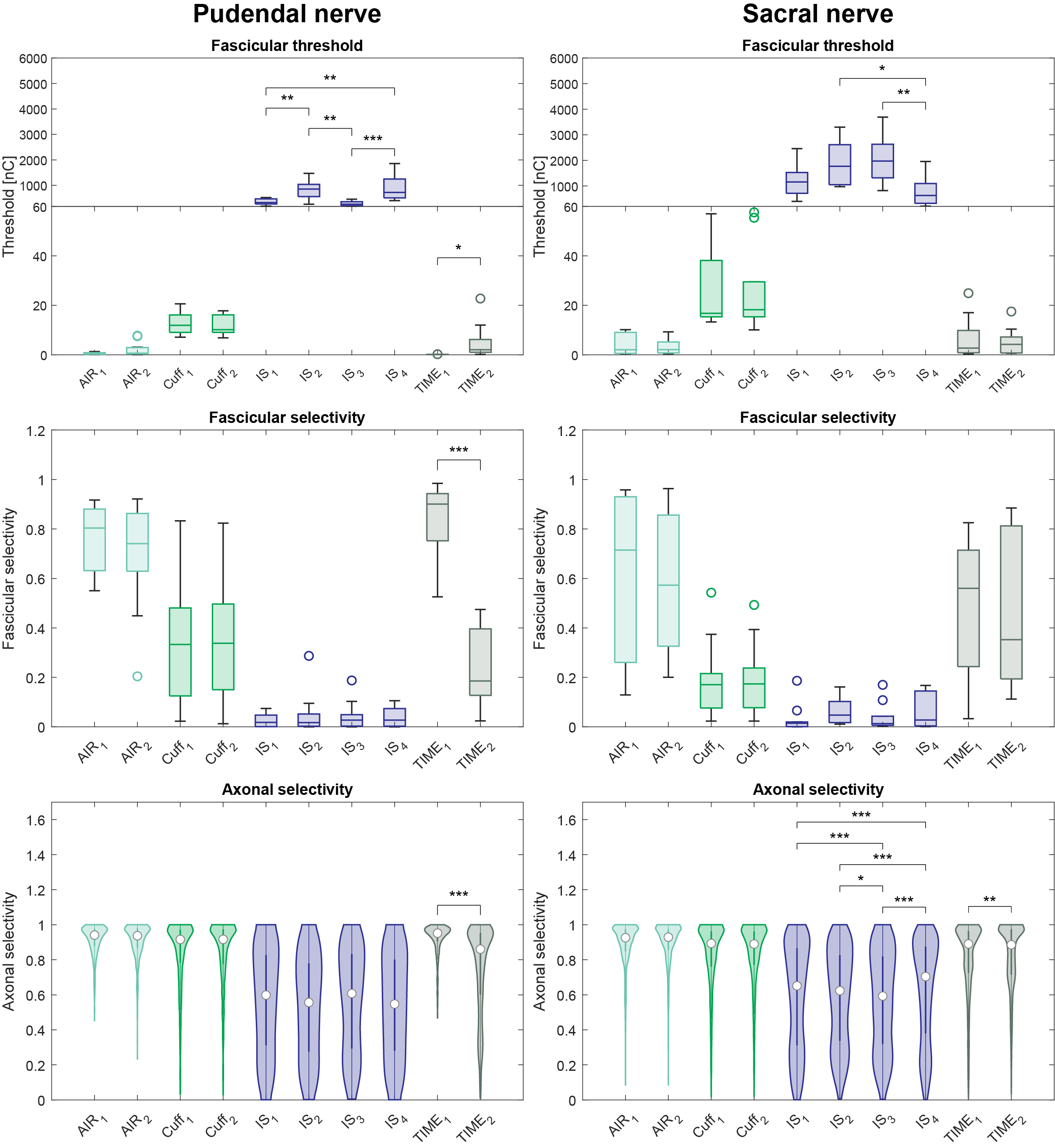

Supplement: S2 Fig — The star-signs label significant differences within electrode type among placements, computed by one-way ANOVA tests with Bonferroni correction for multiple comparisons. (PNG) [file pcbi.1011184.s003.png]

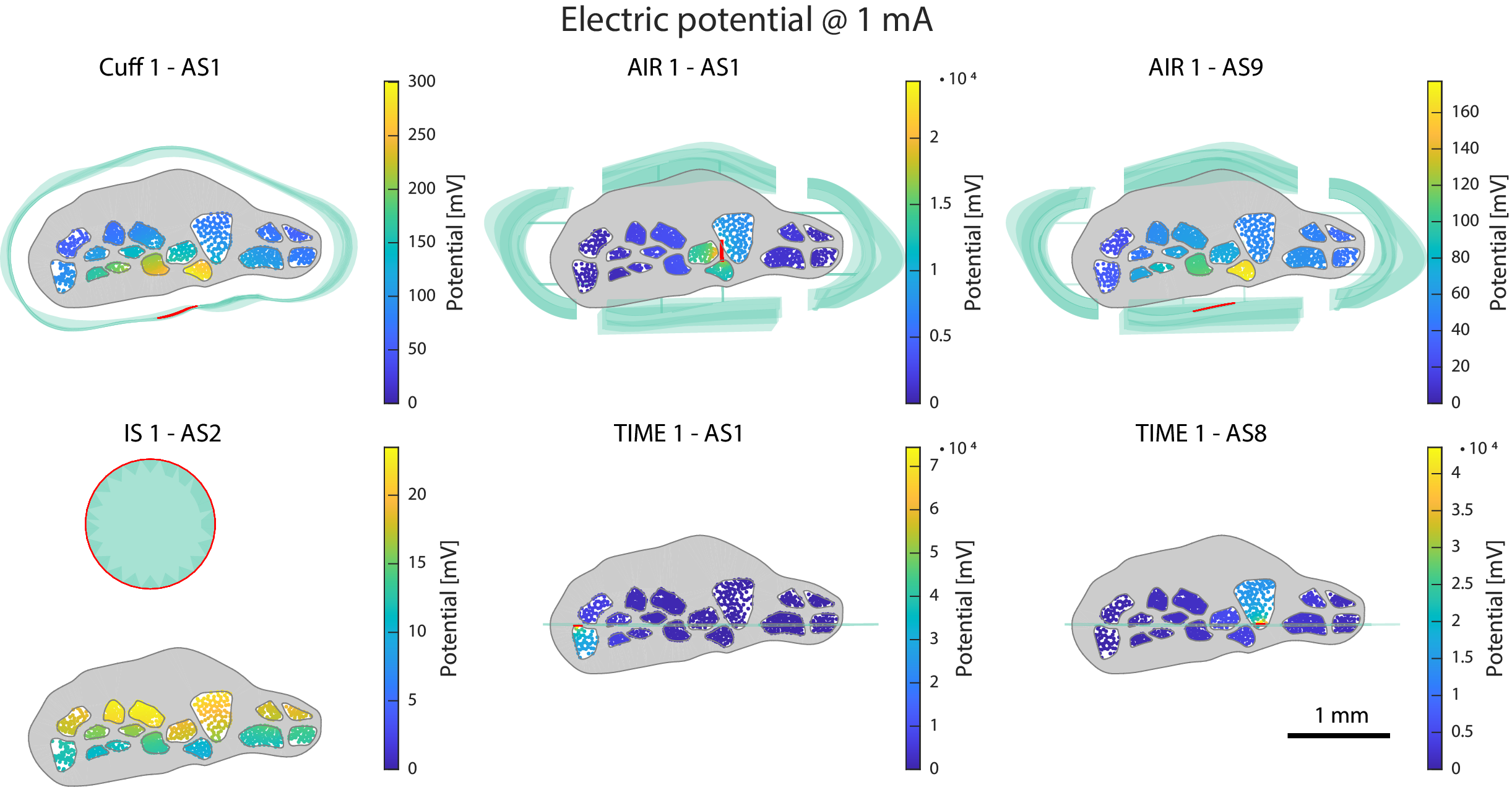

Supplement: S3 Fig — Obtained at a reference injected current of 1 mA for a selection of different active sites of InterStim, TIME, and AIR electrodes. (PNG) [file pcbi.1011184.s004.png]

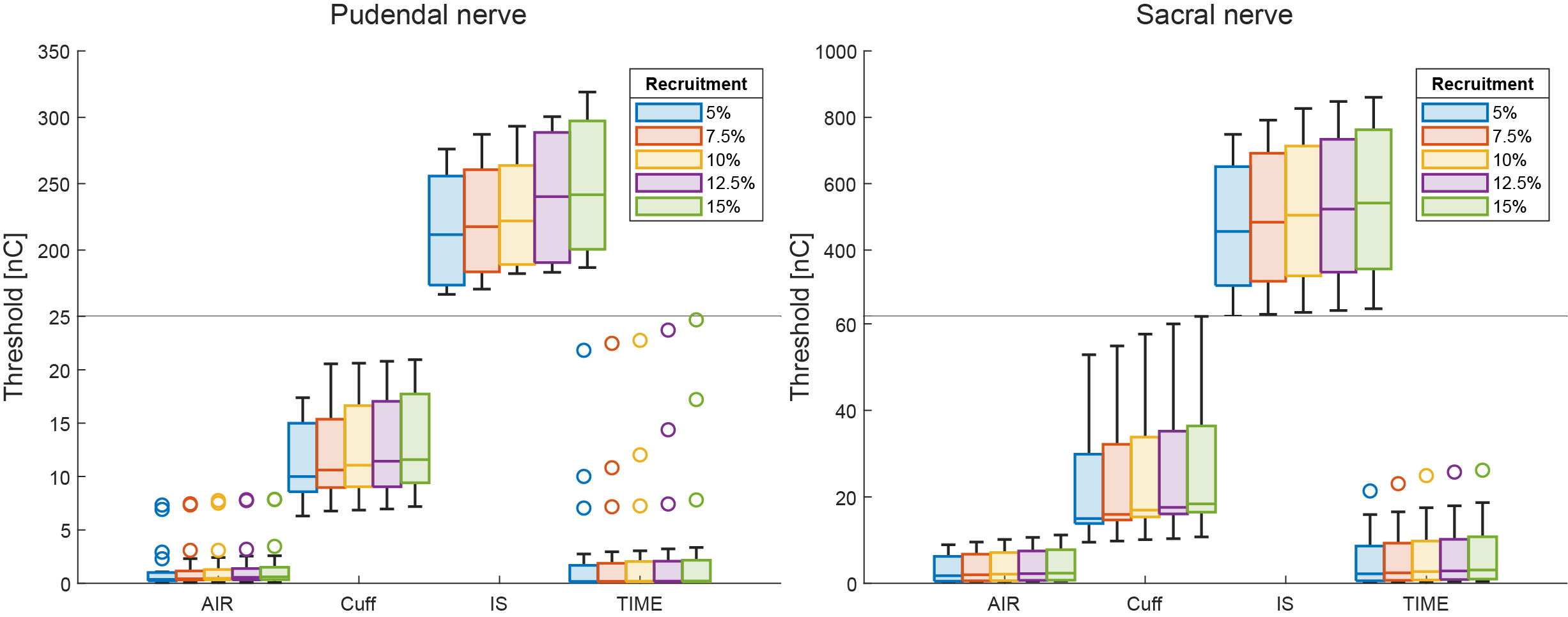

Supplement: S4 Fig — All electrode placements and fascicles are grouped. It can be observed how the relative performance of the four electrode types does not appreciably depend on the target recruitment level. (PNG) [file pcbi.1011184.s005.png]
